# Methods and impact of engagement in research, from theory to practice and back again: early findings from the Patient-Centered Outcomes Research Institute

**DOI:** 10.1007/s11136-017-1581-x

**Published:** 2017-05-12

**Authors:** Laura Forsythe, Andrea Heckert, Mary Kay Margolis, Suzanne Schrandt, Lori Frank

**Affiliations:** 1 PCORI, Washington, DC USA; 2 Arthritis Foundation, Atlanta, GA USA

**Keywords:** Patient engagement, Patient-centered outcomes research, PCORI, CER

## Abstract

**Purpose:**

Since 2012, PCORI has been funding patient-centered comparative effectiveness research with a requirement for engaging patients and other stakeholders in the research, a requirement that is unique among the US funders of clinical research. This paper presents PCORI’s evaluation framework for assessing the short- and long-term impacts of engagement; describes engagement in PCORI projects (types of stakeholders engaged, when in the research process they are engaged and how they are engaged, contributions of their engagement); and identifies the effects of engagement on study design, processes, and outcomes selection, as reported by both PCORI-funded investigators and patient and other stakeholder research partners.

**Methods:**

Detailed quantitative and qualitative information collected annually from investigators and their partners was analyzed via descriptive statistics and cross-sectional qualitative content and thematic analysis, and compared against the outcomes expected from the evaluation framework and its underlying conceptual model.

**Results:**

The data support the role of engaged research partners in refinements to the research questions, selection of interventions to compare, choice of study outcomes and how they are measured, contributions to strategies for recruitment, and ensuring studies are patient-centered.

**Conclusions:**

The evaluation framework and the underlying conceptual model are supported by results to date. PCORI will continue to assess the effects of engagement as the funded projects progress toward completion, dissemination, and uptake into clinical decision making.

**Electronic supplementary material:**

The online version of this article (doi:10.1007/s11136-017-1581-x) contains supplementary material, which is available to authorized users.

## Introduction

Best practice in health-related quality of life (HRQL) assessment evolved to require inclusion of patient input in the development of measures [1], and for many conditions, patient self-report is the standard for assessment of disease and treatment impact [2]. Over the last four decades, the role of the patient in health services research has also expanded. Qualitative and quantitative methods for incorporating the “patient voice” in research are now established [3]. Patients have become increasingly active partners in research, with contributions that extend beyond participation as study subjects. Patient-based advocacy in health research was energized by the ACT-UP movement in the 1980s, changing the norm from investigator-only control of research toward models of shared control. Disability rights activists contributed to this changing research norm through the 1990s, bringing the concept of “nothing about us without us” to public policy and research-based advocacy [4].

The advocacy movement overtly combined political action aims with research, and community-based participatory research emerged as a new model of health research [5]. In the early 2000s, best practice in health outcomes research, which included capturing the patient perspective directly from patients, coalesced with the ethos of patient engagement in research in roles beyond that of study participation only. At the same time, the rise of formal health technology assessment (HTA) programs in the UK, Germany, and elsewhere was coupled with a focus on healthcare stakeholder buy-in in the process and outcomes of HTA, leading to new models of inclusion of patients and others involved in health care. During this time, several countries, including the UK, Canada, Germany, France, and Sweden, initiated or expanded patient and public involvement programs [6–10]. While several hallmarks of HTA have been explicitly rejected in the US, patient and stakeholder engagement have been increasingly embraced as worthwhile [10–12]. The establishment of the Patient-Centered Outcomes Research Institute (PCORI) in 2010 to fund comparative clinical effectiveness research (CER) energized the focus on engagement of patients and other stakeholders as research partners as a new way of pursuing clinical research. PCORI recognizes that in addition to patients, relevant stakeholder groups include clinicians, hospitals and health systems, industry, training institutions, healthcare purchasers, payers, and policymakers. PCORI’s requirement, unique for the large US funders of clinical research, that funded projects engage patients and other stakeholders as partners in the production of the evidence, presents a novel opportunity to address the question of how partner engagement impacts research.

Even in countries in which engagement has been incorporated into health research and HTA for years (for example, Canada, UK), little is known about the specific effects that engaging research partners have on the research process or its outcomes, and collecting this evidence is part of the planning for CIHR and NIHR [13, 14]. The current evidence base is recognized as extremely limited thus far (see for example [15]).We review the evaluation framework developed to guide understanding of PCORI’s work and a conceptual model of patient-centered outcomes research (PCOR) [16], and we present quantitative and qualitative findings from the PCORI experience in partner-engaged research to describe engagement in PCORI projects and characterize its effects. Results are compared against the evaluation framework and conceptual model. Implications for ongoing evaluation of engagement in research are presented.

## Methods

### Conceptual and practical basis for research engagement questions

**Fig. 1:**
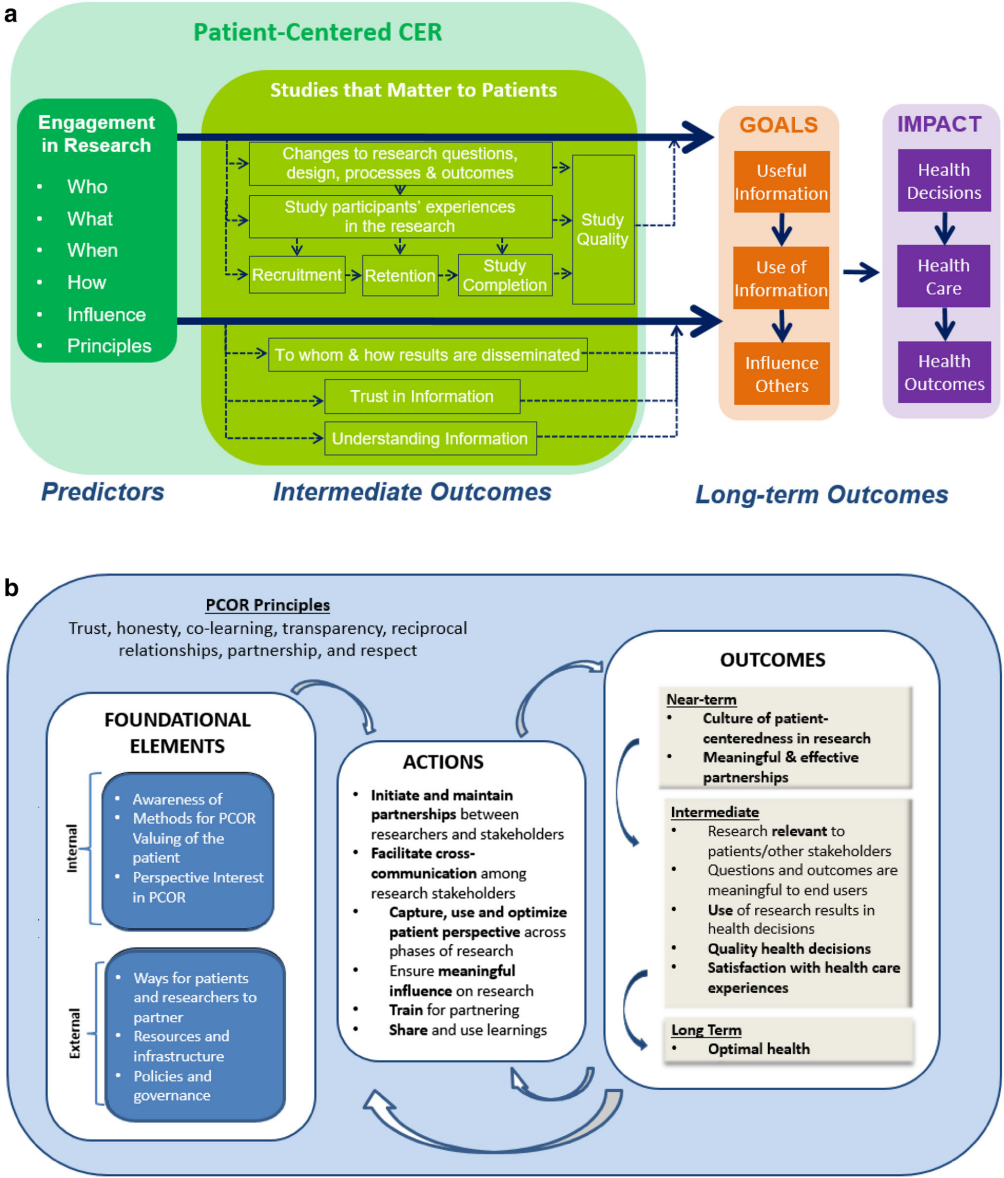
**a** PCORI evaluation framework for engagement in research. Note: to view the full evaluation framework regarding all of PCORI’s work, see http://www.pcori.org/research-results/evaluating-our-work/planning-our-evaluation-reporting-results. **b** Conceptual model of patient-centered outcomes research. Reproduced with permission from Frank et al. [16]

This work was guided by the Evaluation Framework [17], part of PCORI’s evaluation plan, developed with input from several groups representing diverse healthcare stakeholders, including the PCORI Board of Governors, Methodology Committee, and Advisory Panel on Patient Engagement. The full framework addresses all aspects of PCORI’s work and operationalizes questions about PCORI’s work in practice. The section focusing on the impact of engagement in research is the source of the research questions addressed here and is organized into four areas (Fig. 1a):

- description of engagement approaches (who, when, how, etc.), including perceived influence of the research partners and application of the PCOR principles (trust, transparency, honesty, reciprocal relationships, etc.) [16];
- effect of engagement on research processes and intermediate outcomes reflective of studies that matter to patients (e.g., research questions, outcomes selected, study design, dissemination of results);
- longer-term effects of engagement on achievement of PCORI’s strategic goals (http://www.pcori.org/about-us/what-we-do/pcoris-strategic-plan) to increase the quantity and quality of useful information for health decision making, speed uptake of evidence-based information in health decision making, and influence other research to be more patient-centered; and
- impact of engagement in research on better health (health decisions, health care, health outcomes).

The conceptual model of PCOR [16] Fig. 1b specifies foundational elements, actions, and outcomes relevant for patient-centered outcomes research and provides a theoretical foundation for the evaluation framework. The qualitative and quantitative findings presented here provide a check on the accuracy of that model.

**Fig. 2:**
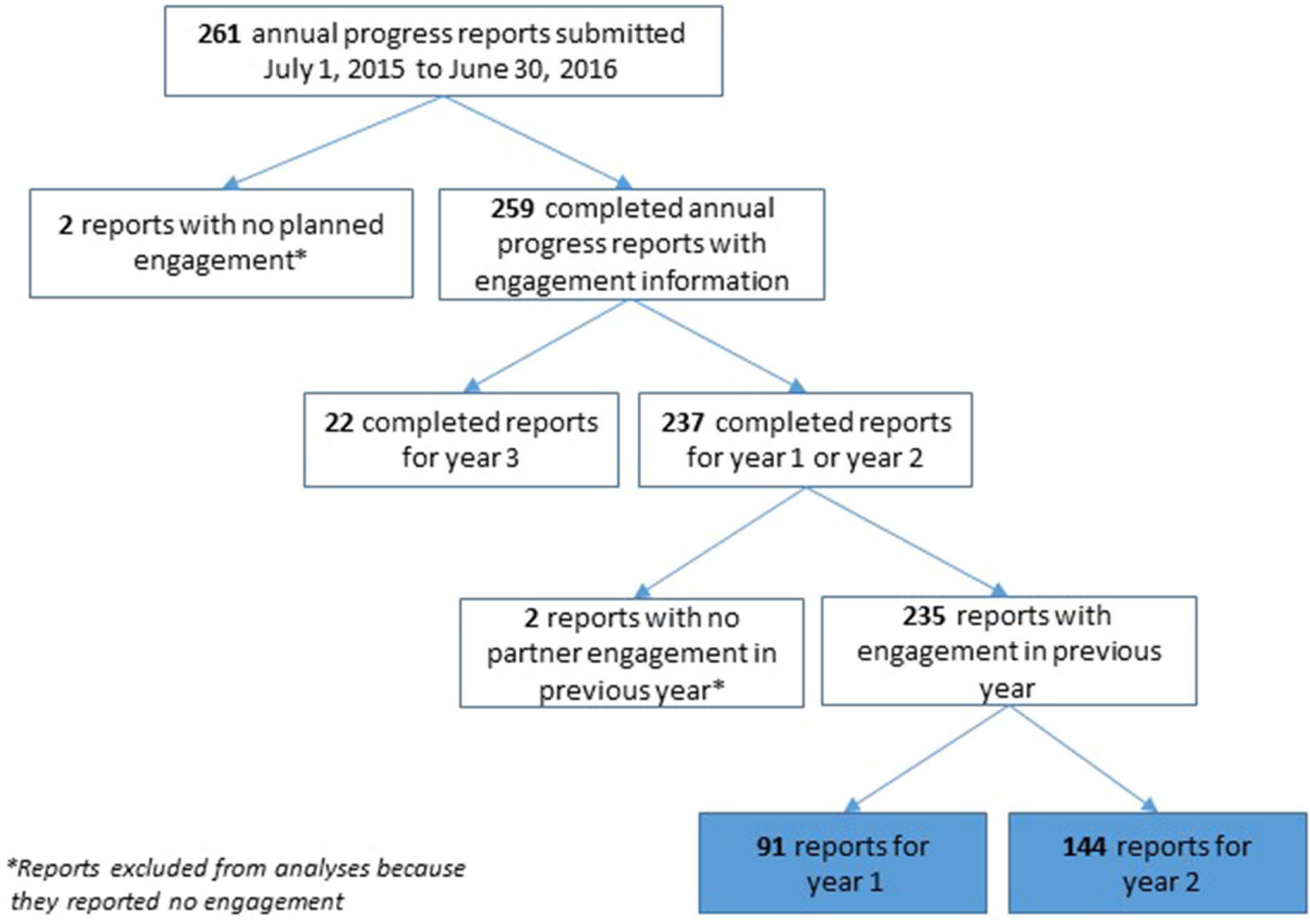
Investigator report sample

### Sample and data collection

From the first funding cycle in December 2012 to June 30, 2016, PCORI funded 434 projects, most for $3 M each and 3 years, but 11 projects were 3- to 5-year “targeted” projects funded for approximately $7 M each, and three pragmatic clinical studies were funded for approximately $12 M each and 5-year duration.

As part of annual project progress reporting, PCORI investigators answer closed- and open-ended questions about their experiences with patient and stakeholder engagement in their PCORI-funded projects. Responses are not anonymous. Data in these analyses are from Year 1 or Year 2 progress reports submitted from July 1, 2015 to June 30, 2016 (reports from the third year of the project are excluded due to small sample size) (Fig. 2). Four projects (<1.5% of sample) reported no engagement and were excluded (two projects that did not include engagement in their research plan and two projects that did not engage with partners during the reporting period).

**Fig. 3:**
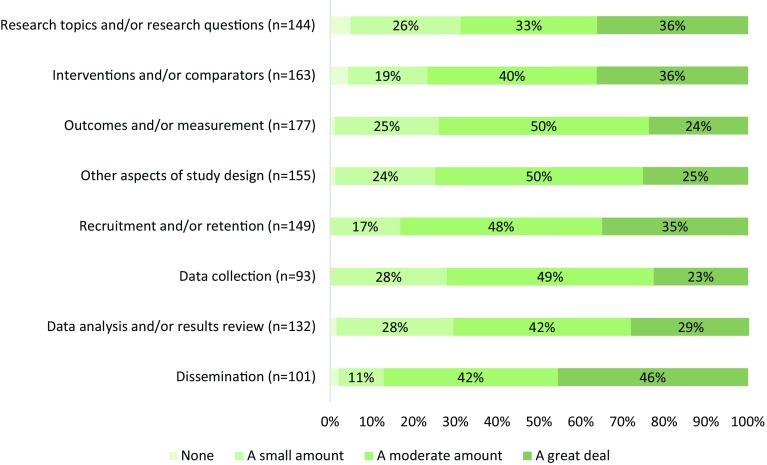
Ratings of partner influence across study phases (investigator-reported)

Upon completion of each annual progress report, investigators are asked to nominate up to 10 patient and other stakeholder research partners to provide feedback on their experience with the project. Partners answer closed- and open-ended items about engagement via the Ways of Engaging-Engagement Activity Tool (WE-ENACT), offered via web survey with an option to complete via phone interview. PCORI emails invitations to partners, with up to three e-mail reminders. Data included in the analyses are from partner reports on their engagement in Year 1 or Year 2 of projects, collected from July 1, 2015 to June 30, 2016. Unlike investigator data, research partner completion of the WE-ENACT is voluntary, so the research partner sample represents a smaller pool of funded projects. MaGil Institutional Review Board approved the protocol for collection of information from research partners and for secondary analysis of project or administrative data.

Although more than half (53%) of the projects in this sample include reports by both the investigators and partners, within-project comparisons between investigators and partners are not made for several reasons. Projects represented in the investigator data may not be represented in the partner data (overall, about two-thirds of investigators nominate partners); partner reporting is voluntary (overall 51% response rate); and due to the time lag between partner nominations and partner reporting, reports from those nominated near the end of the sampling timeframe may not have been available at the time of this analysis. Multiple partners may report on a single project (range 1–8). Partners report on their individual participation and influence and, while most participate in multiple phases of the project, investigators are responsible for the entirety of the project and report on the contributions of all partners collectively.

### Measures

The closed- and open-ended engagement items of the annual progress report for PCORI investigators and the WE-ENACT for research partners (Appendix A & B, online) were developed by PCORI staff based on past data collection efforts [18], PCORI’s Evaluation Framework [17], a conceptual model of PCOR [16], and the published literature. Over time, changes have been made to both data collection tools based on cognitive testing, feedback from investigators and partners, PCORI needs, and standard survey practices (e.g., retiring items that have reached saturation, resting and rotating items to minimize respondent burden, and adding/modifying items to capture new information).

Investigators report on multiple aspects of partner engagement including the communities represented by research partners, the study phases in which partners are engaged, and engagement approaches used. For each study phase with partner engagement, investigators rate the influence of partners (on a 4-point Likert scale from “None” to “A great deal”) and complete an open-ended item on partner engagement activities and the effect of these activities (“Describe what patients and/or other stakeholders actually did and any impact this had on the project”). Investigators also quantitatively rate the partner influence on how the team works together and on research projects other than the specific PCORI-funded project.

Each research partner who completes the WE-ENACT reports the primary community he/she represents on the project, the study phases in which he/she has been engaged, and demographic information. For each relevant study phase, the partner is asked to “Describe what you did and how it made a difference.”

### Analysis

Cross-sectional analyses were conducted separately for the investigator and partner samples. Closed-ended item responses from investigators were quantitatively analyzed using descriptive statistics (e.g., proportions, means). Open-ended item responses from both investigators and partners were analyzed via content and thematic analysis [19, 20]. Hierarchical codebooks were developed using deductive (generated through prior work) and inductive approaches (based on the current analytic samples). Codes were applied to relevant text using NVivo v11 software. To ensure coder agreement, three coders independently coded 10% of the same data and met to reconcile discrepancies. Coded text occurrence queries were generated using NVivo. A frequentist approach to explain the patterns in the data and establish the prominence of themes was deemed appropriate considering the highly structured data collection tools [21]. The minimum threshold for theme identification was 10% of the relevant responses from both respondent samples. Results are presented separately for the two main aims: description of engagement and characterization of effect of engagement.

## Results

### Investigator report sample

These analyses include 235 reports from investigators: 91 reports on Year 1 and 144 reports on Year 2 (Fig. 2). The projects represent PCORI’s five program areas (*n* = 221; 94%), including “targeted” projects (*n* = 11; 5%) and pragmatic projects (*n* = 3; 1%). Three quarters of these investigators had 10 or more years of research experience and 52% were male (Table 1). The majority (96%) of the investigators were reporting on their first PCORI award; 42% had previously been principal investigators on more than 10 research studies awarded by other funders.

**Table 1:** Project reports: investigator characteristics

Characteristic	Year 1 reports (*n* = 91)	Year 2 reports (*n* = 144)	Total (*N* = 235)
Gender (*n*, %)			
Female	44 (48%)	68 (47%)	112 (48%)
Male	47 (52%)	76 (53%)	123 (52%)
Research experience^a^ (*n*, %)			
0–4 years	7 (8%)	5 (4%)	12 (5%)
5–9 years	16 (18%)	29 (20%)	45 (19%)
10+ years	68 (75%)	108 (76%)	176 (76%)
Missing	0	2	2
Previous projects as PI^b^ (*n*, %)			
0	3 (3%)	1 (<1%)	4 (2%)
1–5	28 (31%)	53 (37%)	81 (35%)
6–10	25 (27%)	27 (19%)	52 (22%)
11–15	14 (15%)	16 (11%)	30 (13%)
16–20	7 (8%)	14 (10%)	21 (9%)
21+	14 (15%)	32 (22%)	46 (20%)
Missing	0	1	1

^a^Based on question: How many years of research experience do you have related to this field of research?

^b^Based on question: Approximately how many grants/contracts have you had funded as the PI or project lead?

### Partner report sample

These analyses include 123 reports from Year 1 and 137 reports from Year 2, a total of 260 reports from partners, from 124 different projects, with one to seven partners reporting per project. (mean 2.1 ± 1.3). Partners in this reporting sample were mostly female (70%) and white (78%), mean age of 54 (±13) (Table 2). The partners in the projects most commonly represented the patient/consumer (29%), caregiver (12%), or clinician (15%) communities.

**Table 2:** Project reports: partner characteristics

Characteristics	Year 1 reports (*n* = 123)	Year 2 reports (*n* = 137)	Total (*N* = 260)
Age (mean ± SD years)	55 (±13) (*n* = 115)	54 (±13) (*n* = 128)	54 (±13) (*n* = 243)
Gender (*n*, %)			
Female	79 (68%)	96 (73%)	175 (70%)
Male	37 (32%)	36 (27%)	73 (29%)
Transgender	1 (<1%)	0 (0%)	1 (<1%)
Missing	6	5	11
Race (*n*, %)			
American Indian/Alaska Native	0 (0%)	3 (2%)	3 (1%)
Asian	4 (3%)	5 (4%)	9 (4%)
Black or African American	12 (10%)	20 (15%)	32 (13%)
Native Hawaiian or other Pacific Islander	1 (<1%)	1 (<1%)	2 (<1%)
White	95 (80%)	98 (75%)	193 (78%)
Other	7 (6%)	3 (2%)	10 (4%)
Missing	4	7	11
Ethnicity (*n*, % Hispanic/Latino)	7 (6%) (*n* = 118)	5 (4%) (*n* = 131)	12 (5%) (*n* = 249)
Primary partner community represented (*n*, %)			
Patient/consumer	35 (32%)	37 (28%)	72 (29%)
Clinician	18 (16%)	14 (11%)	32 (13%)
Caregiver/family member of patient	12 (11%)	18 (14%)	30 (12%)
Patient/caregiver advocacy organization	17 (16%)	7 (5%)	24 (10%)
Community-based organization	5 (5%)	12 (9%)	17 (7%)
Subject matter expert	7 (6%)	8 (6%)	15 (6%)
Clinic/hospital/health System representative	5 (5%)	7 (5%)	12 (5%)
Payer (public or private insurance)	0 (0%)	4 (3%)	4 (2%)
Policy maker (government official)	0 (0%)	2 (2%)	2 (<1%)
Other^a^	11 (10%)	23 (17%)	34 (14%)
Missing	14	5	18
Educational attainment (*n*, %)			
Less than high school	0 (0%)	1 (<1%)	1 (<1%)
High school graduate or GED	2 (2%)	3 (2%)	5 (2%)
Post high school training other than college (vocational or technical)	3 (3%)	4 (3%)	7 (3%)
Some college	16 (13%)	25 (19%)	41 (16%)
College graduate	28 (23%)	31 (23%)	59 (23%)
Postgraduate	71 (59%)	69 (52%)	140 (55%)
Missing	3	4	7
Previously partnered on other research project^b^ (*n*, % yes)	64 (54%) (*n* = 119)		
Previously partnered with current investigators^b^ (*n*, % yes)	46 (42%) (*n* = 109)		
Time worked with current investigators^b,c^ (mean ± SD)	4.3 (± 3.0) (*n* = 45)		
Study phase(s) in which engaged			
Researcher understanding of patient and stakeholder needs	96 (86%)	102 (77%)	198 (81%)
Research topics and/or research questions	43 (38%)	37 (28%)	80 (33%)
Interventions and/or comparators	44 (39%)	34 (26%)	77 (32%)
Outcomes and/or measurement	62 (55%)	56 (42%)	118 (48%)
Recruitment: Training research staff on how to recruit and work with patients	35 (31%)	23 (17%)	58 (24%)
Recruitment and retention: Finding and/or retaining participants	49 (44%)	43 (33%)	92 (38%)
Data collection	23 (21%)	20 (15%)	43 (17%)
Data analysis and/or results review	39 (35%)	56 (42%)	95 (39%)
Data application to real world settings	34 (30%)	42 (32%)	76 (31%)
Dissemination	22 (20%)	40 (30%)	62 (25%)
Missing	11	5	16

^a^Includes: Advisory panel member; Community-based organization and free clinic/pharmacy; Chair, parent advisory board; Clinical informaticist; Clinical researcher; Clinical social worker; Community advisor; Community partner intermediary and cultural broker; Disparity expert; Executive director of patient foundation; Long-term and post-acute care provider trade association; Parent; Parent and leader of advocacy organization; Patient advisor x 2; Patient advisor/co-author; Patient advocate x 2; Patient and caregiver; Patient and research advocate; Patient and subject matter expert; Patient/consumer/caregiver/family member of patient; Patient family and child advocate; Peer group facilitator; Practice-based co-PI; Previously a patient; Professional society representative; Project consultant x 2; Research assistant with lived experience; Research expert x 2; Survivor of child abuse

^b^Item only asked at Year 1

^c^Item only asked of respondents who indicated they previously partnered with the current investigators

### Description of engagement

#### Quantitative findings

A majority of the investigators reported engaging with research partners that were patients (88%) and/or clinicians (89%), and more than half reported engaging with clinic or health system representatives (60%), patient or caregiver advocacy organizations (57%), and caregivers (51%) (Table 3). Investigators reported engaging an average (±SD) of 4.9 ± 2.0 communities (range 1–11). Common approaches to engaging research partners were via advisory groups (82%), and as research team members (81%); fewer investigators endorsed using opinion polls/interviews/surveys (39%). More than half (56%) of the investigators who reported engaging partners as research team members identified them as engaging at the most active level, as co-investigators on the project. On average, investigators reported using 2.6 ± 1.1 different approaches to engaging partners (range 1–5). Investigators reported that partners were engaged across eight possible study phases (from identifying research topics to disseminating research results; mean 4.9 ± 1.9 phases, range 1–9 when “other” is included). Outcomes and measurement identification were the most common phase with engagement (75%). As expected, the proportions of investigators reporting that partners were engaged in the later aspects of a project, including data collection, data analysis/results review, and dissemination were higher for Year 2 reports.

**Table 3:** Characteristics of engagement in research (investigator-reported)

	Year 1 reports (*n* = 91)	Year 2 reports (*n* = 144)	Total (*N* = 235)
Partner communities engaged^a^			
Clinician	83 (91%)	126 (88%)	209 (89%)
Patient/consumer	82 (90%)	125 (87%)	207 (88%)
Patient/caregiver advocacy organization	56 (62%)	84 (58%)	140 (60%)
Clinic/hospital/health System representative	53 (58%)	81 (56%)	134 (57%)
Caregiver/family member of patient	43 (47%)	77 (53%)	120 (51%)
Subject matter expert	43 (47%)	78 (54%)	121 (51%)
Training Institution representative (non-research health professions educator)	15 (16%)	22 (15%)	37 (16%)
Policy maker (government official)	10 (11%)	28 (19%)	38 (16%)
Payer (public or private insurance)	13 (14%)	22 (15%)	35 (15%)
Life sciences industry representative	2 (2%)	9 (6%)	11 (5%)
Purchaser (small or large employers)	0 (0%)	5 (3%)	5 (2%)
Other^b^	26 (29%)	68 (47%)	94 (40%)
Approaches to engaging partners^a^ (*n*, %)			
Patient/stakeholder research team members	74 (81%)	118 (82%)	192 (82%)
Team members as co-investigators^c^	44 (59%)	63 (53%)	107 (56%)
Advisory groups	72 (79%)	123 (85%)	195 (83%)
Opinion polls or interviews	39 (43%)	53 (37%)	92 (39%)
Other^d^	4 (4%)	13 (9%)	17 (7%)
Study phases in which partners were engaged^a^ (*n*, %)			
Research topics and/or research questions	54 (59%)	90 (63%)	144 (61%)
Interventions and/or comparators	62 (68%)	101 (70%)	163 (69%)
Outcomes and/or measurement	71 (78%)	106 (74%)	177 (75%)
Other aspects of study design	61 (67%)	94 (65%)	155 (66%)
Recruitment and/or retention	53 (58%)	97 (67%)	150 (64%)
Data collection	29 (32%)	64 (44%)	93 (40%)
Data analysis and/or results review	34 (37%)	98 (68%)	132 (56%)
Dissemination	24 (26%)	77 (53%)	101 (43%)

^a^Not mutually exclusive

^b^Includes biostatisticians, case managers, clinical investigators, community health worker organizations, community-based organizations, community residents, dietitians, educational institutions, National Institutes of Health, nurses, professional organizations/societies, regulatory/compliance professionals, support group organizations, and technology advisors

^c^Asked only to those reporting patient or stakeholder partner research team members

^d^Includes “conference presentations”, “conversations”, “peer buddies”, “pilot study participants”, and “webinars”

#### Qualitative findings

Investigators and partners described a wide range of engagement activities (Table 4). Partners commonly described how they s*hared personal perspectives* in early study phases. These perspectives were grounded in partners’ lived experiences (e.g., living with or caring for someone with a health condition among patients and caregiver research partners) and professional expertise (e.g., priorities for clinical care among clinician partners or reimbursement decisions among payer partners), and provided insights on how projects could best address the needs and preferences of the priority patient population(s) and thereby ensure patient-centeredness. For example, one patient/consumer was able to share “how different cultures, genders, and age groups of patients value medical communications and attribute meaning to the end of life” (see Table 4 for additional examples).

**Table 4:**
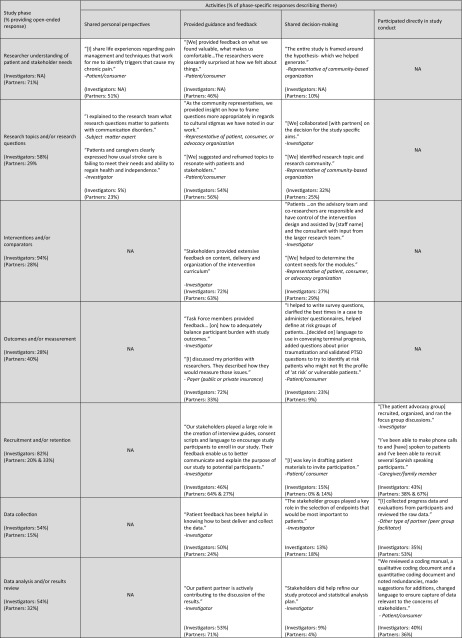

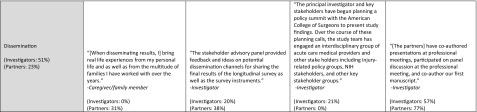
Partner engagement activities—illustrative quotations by study phase (*N* = 235 investigator reports; *N* = 260 partner reports)

Both investigators and partners commonly reported that partners *provided guidance and feedback* or *shared decision* *making* about research questions, design, processes, materials, and outcomes. Across relevant phases, between 20 and 72% of investigator and partner responses described how partners *provided guidance and feedback*. Fewer described how partners *shared decision* *making* (but exceeded the 10% theme identification threshold). Both respondent groups also described how partners *participated directly in study conduct* (from 36 to 77% of responses in relevant phases). Specifically, partners participated in study participant recruitment (e.g., speaking directly to patients, training research team staff to interact with specific patient groups) and data collection (e.g., conducting interviews, co-facilitating focus groups, administering surveys to study participants, tracking study participant visits). Despite none of the projects being complete yet, investigators and partners reported partner involvement in dissemination activities (e.g., co-presenting at scientific meetings, developing manuscripts, determining avenues to share findings, writing newsletters, participating in media interviews, speaking with public health officials about the project).

### Effects of partner engagement

#### Quantitative findings

Investigators indicated that research partners exerted influence in multiple ways, with more than two-thirds indicating at least a moderate influence (Fig. 3). Most investigators (73%) indicated that partners had a moderate or great deal of influence on how the team works together. More investigators noted influence at Year 2 relative to Year 1 on research projects beyond the current PCORI-funded (53 vs. 34% rating of moderate or greater influence, (*x* ^2^ = 8.08, *p* < 0.01).

#### Qualitative findings

As a result of partner engagement, multiple aspects of the projects were refined and made more patient-centered (Table 5). Across relevant phases, between 11 and 52% of investigator and partner responses described *enhanced patient*-*centeredness of study processes and outcomes*, and 20–81% described *enhanced study design, conduct, or efficiency*. As described below, research partners had an impact on selection of research topics and/or research questions, interventions and/or comparators, and outcomes and/or measures used. Both investigators and research partners describe participant recruitment and retention and data collection as more efficient as a result of partner engagement. Although few projects in this sample were nearing completion, there is evidence of research partner influence on affecting data analysis and/or results review and dissemination of study results. These qualitative findings correspond to the investigators’ quantitative report of influence of research partners across study phases (Fig. 3).

**Table 5:**
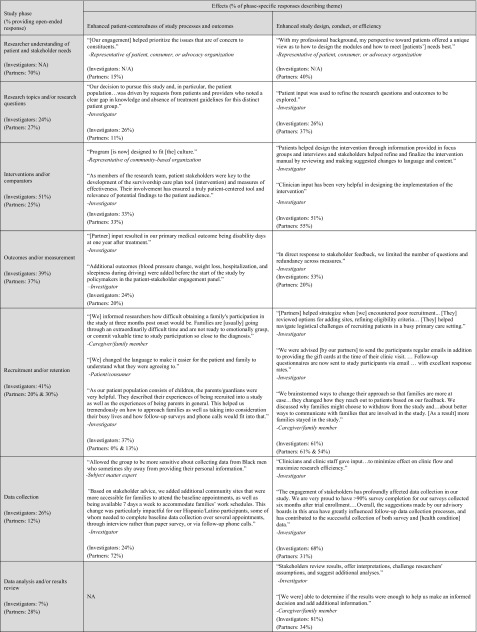

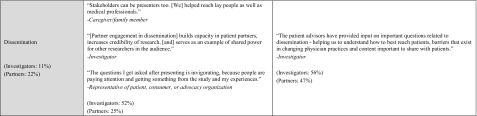
Effects of partner engagement—illustrative quotations by study phase (*N* = 235 investigator reports; *N* = 260 partner reports)

Both investigators and partners reported that partner input confirmed the importance of research topics they were pursuing, inspired pursuit of specific research questions, and/or refined the research questions to be relevant and aligned with patient or other stakeholders’ priorities. For example, one patient/consumer noted “Anxiety became a study topic when it had not been considered before” (see Table 5 for additional examples). Partners also contributed to refining interventions and/or comparators to be more patient-centered, adapting materials or interventions to be culturally/linguistically appropriate, and modifying the intervention to be less burdensome to participants. Partner contributions to outcomes and/or measurement phase include selection of specific primary and secondary outcomes that matter to patients and other information users, and identification and/or refinement of measures of these constructs. Partners often noted that outcomes of interest to them would have been otherwise overlooked and remained unmeasured.

Investigators and partners reported changes to recruitment strategies such as adding or changing recruitment locations, refining inclusion/exclusion criteria, and use of culturally appropriate ways to recruit specific populations. Partner input shaped materials and consent forms (e.g., streamlining, adding more information about risks and benefits). Partners also contributed to participant retention through guidance on the best ways to communicate and suggesting new modes of data collection. Both investigators and partners recognized that partner input contributes to greater perceived value of trial participation among enrolled patients/caregivers, enhanced enrolment rates, and/or improved retention throughout project follow-up periods. Effects of partner contributions on data collection include selection of specific modes of data collection (e.g., electronic vs. phone), informed decisions about timing such as the appropriate follow-up periods, changes as part of clinic work flow, and increased sensitivity around data collection (e.g., insights on why racial/ethnic minorities may be hesitant to share personal information).

While these projects are not complete, early signals indicate effects of engagement on data analysis and/or results review, dissemination, new ways to share results, new audiences to reach, improved communication with different audiences, and increasing credibility of the findings.

## Discussion

PCORI’s requirement that awardees engage patients and/or other stakeholders as partners in the research it funds presents a unique opportunity to describe engagement in research as it is implemented across a large portfolio of CER. PCORI’s effort to understand engagement in research is a unique, systematic data collection initiative, using data from both investigators and their research partners. This quantity of rich information on the engagement experience from multiple perspectives is not available from any other research funder.

PCORI investigators reported engaging a greater number of different types of stakeholder communities in more phases of the project and through more active approaches (e.g., partner co-investigator) than has been previously documented in the literature. For example, Concannon et al. found that most projects in the published literature that reported involving research partners engaged with patients, about half engaged with clinicians, and a few involved other stakeholders [22]. In contrast, nearly all the PCORI reports in this sample indicated engagement with both patients and clinicians. Engagement with caregivers, patient and caregiver advocacy organizations, and health systems representatives occurred in more than half of the reports. Inclusion of these diverse perspectives in production of research evidence is unprecedented on this scale and presents the opportunity to learn about the ways in which such inclusion changes research process, research quality, and speed of uptake.

In the data reported here, investigators and partners recognized similar effects of partner engagement, including refinements to research questions, design, study processes, and outcomes selection. These effects may address historic challenges in clinical research that limit the value of research for its end-users [23] by increasing the relevance and importance of the research for those end-users. Both investigators and partners report that engagement aided recruitment and retention, particularly noteworthy given that failures in recruitment and retention are a major factor in clinical trial failure [24]. Improvements in data collection efforts are also noteworthy, and suggest engagement may enhance other strategies to reduce missing data [25]. The findings here reinforce findings from smaller samples that did not include the partner view [8, 26–30].

PCORI expects engagement to inform key aspects of its funded projects but not necessarily every phase of every project. The role of engagement for a given project depends on the project content and context, and goals and past work in that research area. More work is underway to identify optimal engagement models by understanding intensity (e.g., in the number of partner types, phases and methods of engagement,), as well as effects of engagement such as the number and type of outcomes selected for study, recruitment rates, time to study completion, and study quality.

Investigators reported that partners influence the way the team works together. The implications of this should be further explored given the reported challenges of fully including diverse partner types and managing different perspectives [31]. Investigators, particularly those in the second year, also noted that partners influence other projects beyond the PCORI project. More research is needed to determine whether the number of investigators reporting such influence grows as the PCORI projects progress beyond the second year. The investigator ratings of research partner influence suggest a shift from more transactional approaches to engagement (e.g., discrete, one-way interactions) to more relational approaches, and qualitative analyses will continue to aid full understanding. Longitudinal examination will permit capture of the potential for transforming programs of research, affecting researchers’ career trajectories, and changing the culture of how research is conducted [16]. Understanding the challenges of research engagement and strategies for overcoming those challenges is also critical to supporting a culture of engaged research. Although a detailed analysis of challenges and facilitators of engagement is beyond the scope of this paper, both investigators and partners identify key challenges, such as barriers to scheduling and logistics, limits on engagement due to health problems, and difficulty identifying and fully involving diverse partners [31].

Across study phases, investigators report more partner engagement than do partners in this sample. This may be due to limited visibility responding partners have of the extent of partner engagement in the project, since partners are asked to report on their individual contributions and many are engaged in limited parts of the project, while investigators report on the collective contributions of all partners across the entire project. Further, partners have recognized a need for investigators to more frequently and clearly report back to partners the ways that their input has contributed to the study [31]. While the discrepancy may also reflect over-reporting by investigators, this possibility is mitigated by ongoing relationships between investigators and PCORI project officers.

While this study represents significant advancements in knowledge about engagement in research, several limitations exist. These self-report data require respondents to recall their experiences over the past year, and likely capture the most salient, but not all, effects of engagement. Furthermore, respondents, particularly investigators, may overestimate their positive experiences with engagement given that this information is reported to the research funder. Although similar themes were identified among investigators and partners, partner reports may underreport the impacts of engagement because some research teams are more effective than others at communicating with partners about the effects of their contributions. Furthermore, the partner views reported here may not represent all PCORI research partners; only a subset of projects have partner respondents in the sample, and partners with more positive experiences may have been more likely to respond. The potential selection bias represents an important limitation to interpretation of partner report particularly. Further, the data were collected in English only, and the low proportion of Hispanic respondents suggests that the current sample may not fully represent all target study populations. Demographic differences between respondents and non-respondents are unknown. The data also do not yet include large enough sub-samples for meaningful comparison by stakeholder type. Additionally, although these items were refined through cognitive interviewing, additional measure refinement and item performance evaluation are needed. Self-report is an important source of information about engagement in research, but other complementary methods, including observational approaches, would enhance understanding and overcome limitations of self-report. Moreover, findings may not generalize to studies funded by others under different requirements, in different contexts, and in other fields. Finally, data reported here are based on the first two years of funded projects. Some effects of engagement, such as impact on patient trust in findings, are not measurable until after project completion.

## Conclusion

As PCORI projects mature towards dissemination and implementation of findings, the ultimate measure of the impact of engagement in research will be the usefulness of CER information, the use of that information in clinical decision making, and the impact on better health decisions, health care, and health outcomes, as noted in the PCORI Evaluation Framework. These current findings suggest that PCORI-funded projects are on the path towards desired impacts of engagement in research, with engagement of patients and other stakeholders as partners affecting research questions, design, processes, and outcome selection, as well as recruitment strategies and enrollment rates. Comparing results to the conceptual model of PCOR [16] shows that several hypothesized actions to facilitate PCOR are evident among the projects, including initiating and maintaining research partnerships, capturing and using partner perspectives, facilitating cross-communication with the research team (supported by open-ended feedback), and ensuring meaningful influence (supported by influence ratings) The near-term outcome of a culture of patient-centeredness is supported by the qualitative findings. Ongoing data collection should inform whether longer-term outcomes as specified in the conceptual model (Fig. 1) are realized and the extent to which specific intermediate outcomes specified in the evaluation framework (Fig. 2) are evident. Comparing results to the conceptual model of PCOR [16] shows that several hypothesized actions to facilitate PCOR are evident among the projects, including initiating and maintaining research partnerships, capturing and using partner perspectives, and facilitating cross-communication with the research team.

### Future directions

Future examinations should explore how engagement affects PCORI’s large, multi-site pragmatic clinical studies and other differences based on study type (e.g., interventional vs. observational, treatment comparisons vs. health system comparisons) and population (e.g., for hard to reach populations). Future research is needed to understand the unintended consequences of engagement (e.g., on project budget and timeline) and how to mitigate them, as well as other effects of engagement in research hypothesized in the conceptual model [16], including the effects on partners, investigators, and their institutions in pursuit of establishing a culture of patient-centeredness in research.

Several longer-term questions remain to be addressed as the PCORI-funded projects are completed. The extent of concordance, and causes of any discordance, in views between investigators and research partners bears examination, particularly as such concordance may vary by type of study and engagement approach. Additionally, overcoming the potential selection bias in research partner report requires outreach to more research partners and PCORI is exploring ways to obtain a wider range of perspectives on research engagement from the engaged research partners. Further, understanding differences in the effects of engagement by partner type could ultimately inform strategies for how to engage with different stakeholder communities at various parts of the research process.

The evaluation framework and conceptual model that guide this work are applicable to research in which health care stakeholders are actively engaged. They can guide collection of data beyond PCORI and provide a foundation for the accumulating evidence about engagement in research, providing a means to improve the efficiency and effectiveness of engagement itself. As the evidence base about research engagement expands, the role of engagement in research in improving public health must be re-examined, to support the incorporation of new knowledge into practice through a feedback loop from theory to research and back.

## Electronic supplementary material

Below is the link to the electronic supplementary material.

Supplementary material 1 (PDF 367 kb)

Supplementary material 2 (PDF 199 kb)
