# Supplementary material for: Methods and impact of engagement in research, from theory to practice and back again: early findings from the Patient-Centered Outcomes Research Institute
Source: Qual Life Res. 2017 May 12;27(1):17–31. doi: 10.1007/s11136-017-1581-x (PMC5770504; doi:10.1007/s11136-017-1581-x)
Supplement: Supplementary file 1 — Supplementary material 1 (PDF 367 kb) [file 11136_2017_1581_MOESM1_ESM.pdf]

PCORI Annual Engagement Report

Patient and/or other stakeholder engagement involves having patients and/or stakeholders as partners in research instead of as research subjects only. For example, patients and/or other stakeholders can be involved in activities such as

- Deciding what to study;
- Designing the study;
- Choosing study outcomes;
- Tracking study progress; or
- Sharing study findings

These questions will ask:

- **Who** was engaged in your project (stakeholder communities represented),
- **When** they were involved (which parts of the research process),
- **How** they were engaged (what approaches you used and the level of engagement),
- **How much** influence they had, and
- **What** they did

Report on ***completed*** rather than planned activities ***for the current reporting period***.

For reference only

pcori
PATIENT-CENTERED OUTCOMES RESEARCH INSTITUTE

1. WHO?

a) Which communities were engaged with this PCORI project? Check all that apply. For research partners who may represent more than one community, select the primary perspective the partner brings to your project.

☐ Patient/consumer
☐ Caregiver/family member of patient
☐ Advocacy Organization (e.g., patient/consumer or caregiver advocacy organization)
☐ Clinician
☐ Clinic/ Hospital/ Health System Representative
☐ Purchaser (small or large employers)
☐ Payer (public or private insurance)
☐ Life Sciences Industry
☐ Policy Maker (government official)
☐ Training Institution (non-research health professions educator)
☐ Subject matter expert
☐ Other – *Please describe:*
☐ None (no engagement with patients and/or other stakeholders during the reporting period)

b) **[Answer if Q1a = None]** Engagement may not occur at all time points or may not occur as planned. Which option best describes why engagement with patients and/or other stakeholders has not occurred?

☐ Not part of my research plan
☐ Plans for engagement have been delayed or changed
☐ Other: *(Please describe)*

(For those answering "none" to Q1a, reporting is complete at this time)

2. WHEN?

When in the project were patients and/or stakeholders engaged? *Check all that apply.*

☐ Deciding what the study is about (choosing the research topics or the research question)
☐ Choosing or designing interventions or comparators
☐ Choosing outcomes and deciding how to measure them
☐ Other aspects of study design
☐ Recruiting or retaining study participants
☐ Data collection
☐ Analyzing or reviewing results
☐ Dissemination
☐ Other part of the project- *Please describe:* ___________________________

3. HOW?

a) How did you engage with patients and/or other stakeholders in this research project? *Check all that apply.*

☐ **As Research team member(s):** Consultants, advisors, experts listed as research staff or team members.
☐ Via **Advisory group(s):** Individuals participating in an advisory panel, community advisory board, focus group, community forum, town hall meeting, or other type of group forum to give input on what to study or how to design the project.
☐ **Via Opinion poll(s) or interview(s):** Individuals complete a set of questions to give input on the research process.
☐ **Other:** Please describe: ________________________________

b) **[Answer if Q3a = Research Team Member]** Are any of the patient and/or stakeholder partners serving as Co-Investigators? Co-Investigators typically devote a specified percentage of time to the project and share responsibility for the project with Principal Investigator(s).

☐ Yes
☐ No

c) For each part of the project, which choice best reflects the ways in which patients and/or other stakeholders were engaged?

Definitions:

- **Information:** Researcher(s) describe decisions to patients and/or other stakeholder partners after decisions are made.
- **Consultation:** Patients and/or other stakeholders provide input to researchers that may inform decision-making.
- **Collaboration:** Patients and/or other stakeholders work directly with the researcher, in active partnership, to ensure that their perspectives are incorporated in decision-making.
- **Patient/Stakeholder Direction**: Also known as "user control," patients and/or other stakeholder(s) have control over the research process and the final decision-making.

(Question continued on next page)

For reference only

	Information	*Consultation*	*Collaboration*	*Patient/Stakeholder Direction*
a. Deciding what the study is about	☐	☐	☐	☐
b. Choosing or designing interventions or comparators	☐	☐	☐	☐
c. Choosing outcomes and deciding how to measure them	☐	☐	☐	☐
d. Other aspects of study design	☐	☐	☐	☐
e. Recruiting or retaining study participants	☐	☐	☐	☐
f. Data collection	☐	☐	☐	☐
g. Analyzing or reviewing results	☐	☐	☐	☐
h. Dissemination	☐	☐	☐	☐
i. Other part of the project– Please describe	☐	☐	☐	☐

For reference only

If you have any questions, please email fundedpfa@pcori.org or call 202.370.9500.

pcori
PATIENT-CENTERED OUTCOMES RESEARCH INSTITUTE

4. HOW MUCH?

[For each part of the project selected in Q2] Rate how much influence patients and/or other stakeholders had on each part of this project. By influence, we mean affecting or contributing to decisions or processes related to the project.

	None	*A Small Amount*	*A Moderate Amount*	*A Great Deal*
a. Deciding what the study is about	☐	☐	☐	☐
b. Choosing or designing interventions or comparators	☐	☐	☐	☐
c. Choosing outcomes and deciding how to measure them	☐	☐	☐	☐
d. Other aspects of study design	☐	☐	☐	☐
e. Recruiting or retaining study participants	☐	☐	☐	☐
f. Data collection	☐	☐	☐	☐
g. Analyzing or reviewing results	☐	☐	☐	☐
h. Dissemination	☐	☐	☐	☐
i. Other part of the project Please describe	☐	☐	☐	☐
j. The way the research team and partners work together	☐	☐	☐	☐
k. Research projects other than this specific PCORI-funded project	☐	☐	☐	☐

For reference only

5. WHAT?

[For each part of the project selected in Q2] For each part of the project, describe **what patients and/or other stakeholders actually did** and **any impact** this had on the project.

6. Thank you for your responses to the above survey items. Please feel free to share any other information about engagement in your project – challenges, strategies you've identified to facilitate work with research partners, positive and negative impacts on your work.

For reference only

pcori
PATIENT-CENTERED OUTCOMES RESEARCH INSTITUTE

Partner Nomination

Nominating Patient and/or Stakeholder Partners for Participation

PCORI values input from all research partners. You are invited to provide contact information for patients and/or other stakeholders engaged in your project that would be appropriate for us to contact, so that we can collect information about engagement from their point of view. **Nomination of patient and/or stakeholder partners is completely voluntary.** Patient and/or other stakeholder participation is voluntary. Information obtained from research partners will be used to inform PCORI practices and programmatic improvements.

Please provide the name and contact information of research partners - patients and/or other stakeholders engaged in this research project in roles other than research subjects. Please nominate at least 2 patients and/or other stakeholders. You should provide PCORI only names of patient and/or other stakeholder partners for whom you have obtained appropriate permission to disclose their identity to PCORI.

- Patient/consumer
- Caregiver/family member of patient
- Advocacy Organization (e.g., patient/consumer or caregiver advocacy organization)
- Clinician
- Clinic/ Hospital/ Health System Representative
- Purchaser (small or large employers)
- Payer (public or private insurance)
- Life Sciences Industry
- Policy Maker (government official)
- Training Institution (non-research health professions educator)

We recognize that some partners may have limited access to or comfort with internet based information collection. PCORI will offer a telephone conversation for information collection as needed and will cover any costs this may lead to for the research partner.

First Name	Last Name	Email	Stakeholder Community	Contact by phone, rather than email	Phone Number
			(drop down of choices)		

If you have any concerns about nominating patient and/or stakeholder partners for participation, please explain here or contact us at 202.370.9500.

If you have any questions, please email fundedpfa@pcori.org or call 202.370.9500.

For reference only
